# Dataset of the complete mitogenomes of the mushroom corals Fungiidae

**DOI:** 10.1016/j.dib.2025.111857

**Published:** 2025-07-08

**Authors:** Liwei Li, Zhangwang Lu, Zhiwei Liu, Cheng Liang, Jun Wang, Yan Wang

**Affiliations:** School of Marine Biology and Fisheries, Hainan University, Haikou 570228, PR China

**Keywords:** Mitogenome, Mushroom corals, Fungiidae, Phylogeny, Minisatellite sequences

## Abstract

Twenty-four mitogenomes of the mushroom corals (Fungiidae), representing 18 species from 12 genera, were sequenced and annotated. These mitogenomes exhibit high similarity, each containing the same 13 protein-coding genes (PCGs) and two rRNA genes as other scleractinian corals. Compared to the genes, the intergenic regions (IGRs) are more diverse. Interestingly, minisatellite sequences were identified in the IGRs between *COX1* and *trnM* (IGR*^COX1^*^-^*^trnM^*) and between *ND4* and *rrnS* (IGR*^ND4^*^-^*^rrnS^*) of most Fungiidae mitogenomes. Primarily due to the existence of minisatellites in IGR*^COX1^*^-^*^trnM^*, the length of Fungiidae mitogenomes varies from 16,292 to 17,399 bp. Similar to the phylogenetic tree based on partial COI sequences, Bayesian phylogenetic trees constructed using 13 PCGs, IGR*^COX1^*^-^*^trnM^* and IGR*^ND4^*^-^*^rrnS^* divide Fungiidae into four distinct clades. However, the latter three trees provide a higher resolution of genus- and species-level evolutionary relationships. This mitogenome dataset will be valuable for better understanding the phylogeny of Fungiidae. The diverse IGRs in these mitogenomes may serve as a useful resource for developing Fungiidae DNA barcodes, while the identified minisatellites can facilitate studies on the population biology of Fungiidae.

Specifications Table

**Table:** 

Subject	Biological Sciences
Specific subject area	Bioinformatics, Phylogeny and Evolution, Marine Biology
Data format	Raw, Assembled, Analyzed
Type of data	FASTA: Mitogenome sequence data
Data collection	The Fungiidae samples were collected at Hainan fringe reefs, Xisha Islands and Zhongsha Atoll (all located in the South China Sea), the longitude and the latitude of sample location ranged from 110°53′48″ to 114°47′14″E and from 15°25′38″N to 19°33′9"N, respectively. Genomic DNA was extracted using the Tianamp Marine Animals DNA Kit (TIANGEN, China) and the sequencing was performed on an Illumina NovaSeq 6000 platform with 150 bp paired-end reads length. The reads were assembled into contigs using MitoZ v2.3 and gene annotation was conducted by MITOS web server (http://mitos.bioinf.uni-leipzig.de/). MrBayes v.3.2.7 was used for the Bayesian inference analysis and ETE Tree Browser was used to visualize the phylogeny tree. The minisatellite sequences in mitogenome were screened with MISA software.
Data source location	The Fungiidae specimens were deposited at School of Marine Biology and Fisheries, Hainan University, Haikou 570,228, China (contact person: Yan WANG, ywang@hainanu.edu.cn).
Data accessibility	Repository name: GenBank Data identification number: Accession numbers PQ539056, PQ571311-PQ571325, PQ875194-PQ875201 Direct URL to data: https://www.ncbi.nlm.nih.gov/nucleotide/PQ539056https://www.ncbi.nlm.nih.gov/nucleotide/PQ571311https://www.ncbi.nlm.nih.gov/nucleotide/PQ571312https://www.ncbi.nlm.nih.gov/nucleotide/PQ571313https://www.ncbi.nlm.nih.gov/nucleotide/PQ571314https://www.ncbi.nlm.nih.gov/nucleotide/PQ571315https://www.ncbi.nlm.nih.gov/nucleotide/PQ571316https://www.ncbi.nlm.nih.gov/nucleotide/PQ571317https://www.ncbi.nlm.nih.gov/nucleotide/PQ571318https://www.ncbi.nlm.nih.gov/nucleotide/PQ571319https://www.ncbi.nlm.nih.gov/nucleotide/PQ571320https://www.ncbi.nlm.nih.gov/nucleotide/PQ571321https://www.ncbi.nlm.nih.gov/nucleotide/PQ571322https://www.ncbi.nlm.nih.gov/nucleotide/PQ571323https://www.ncbi.nlm.nih.gov/nucleotide/PQ571324https://www.ncbi.nlm.nih.gov/nucleotide/PQ571325https://www.ncbi.nlm.nih.gov/nucleotide/PQ875194https://www.ncbi.nlm.nih.gov/nucleotide/PQ875195https://www.ncbi.nlm.nih.gov/nucleotide/PQ875196https://www.ncbi.nlm.nih.gov/nucleotide/PQ875197https://www.ncbi.nlm.nih.gov/nucleotide/PQ875198https://www.ncbi.nlm.nih.gov/nucleotide/PQ875199https://www.ncbi.nlm.nih.gov/nucleotide/PQ875200https://www.ncbi.nlm.nih.gov/nucleotide/PQ875201
Related research article	NA

## 1 Value of the Data

• This mitogenome dataset can benefit researchers and students working on Fungiidae systematics, phylogeography, and evolution.
• The diverse IGRs in the mitogenomes would be useful for the development of Fungiidae DNA barcode.
• The minisatellites found in most Fungiidae mitogenomes can be applied to the study of population biology.

## 2 Background

The hard coral family Fungiidae (Dana, 1864) belongs to the phylum Cnidaria, subphylum Anthozoa, class Hexacorallia and order Scleractinia [1]. Species in this family grow in a mushroom-like shape, earning them the common name 'mushroom corals' [2]. Currently, this family comprises 16 genera, exhibiting significant evolutionary divergence and rich species diversity [1]. Fungiidae plays a crucial role in maintaining the balance of coral reef ecosystems [2].

Most fungiid corals consist of large, free-living single polyps. They are distinguished from other scleractinian corals by the presence of compound synapticulae between the septo-costal units [2]. Morphological identification at the genus and species levels primarily relies on differences in life cycles and inconsistencies in skeletal architecture, e.g. the corallum wall, septa, and costae [2,3]. However, coral morphological characteristics respond to environmental changes, and many fungiid coral species exhibit diverse and plastic phenotypic variations [4]. Therefore, a combination of molecular analysis and morphological characteristics is typically used for a more accurate classification of Fungiidae. Commonly used molecular markers include partial sequences of cytochrome oxidase subunit I (COI) [5] and the internal transcribed spacer (ITS) [6,7]. However, the resolution of Fungiidae COI is insufficient. In some cases, COI barcode sequences from different genera, e.g. *Lithophyllon undulatum* (GenBank no EU149887), *Lithophyllon scabra* (EU149894), and *Danafungia horrida* (LC191483), are identical. Regarding ITS, while the high diversity among individuals within a species can compensate for the low species resolution of COI barcodes, it may also obscure or complicate phylogenetic relationships [5]. Additionally, individual coral colonies usually host a high degree of intragenomic ITS variation [8], which may lead to the existence of overlapping peaks in sequencing and result in unreliable sequence alignments [9].

Besides COI, other mitogenome sequences including rRNA genes [10] and intergenic regions (IGR) [11], are also used for the species identification of scleractinian corals. Currently, only four Fungiidae mitogenomes have been published [12]. Here we present a dataset of 24 complete Fungiidae mitogenomes, encompassing 12 of the 16 existing Fungiidae genera, and analyzed the nucleotide diversity of their protein coding genes (PCGs), rRNA genes and IGRs, so as to provide basic information for the development of more reliable DNA barcode in Fungiidae. Interestingly, minisatellite sequences were found in the intergenic regions between *COX1* and *trnM* (IGR*^COX1^*^-^*^trnM^*) and between *ND4* and *rrnS* (IGR*^ND4-rrnS^*) in most Fungiidae mitogenomes. These minisatellites could be valuable for the population genetic studies of Fungiidae [13].

## 3 Data Description

A total of 24 Fungiidae mitogenomes, representing 18 species from 12 genera, were sequenced and annotated (Table 1). These mitogenomes exhibit a high degree of similarity (≥ 96.47 %, Supplementary Table 1). In some cases, the similarity between different genera is greater than interspecific similarity, e.g. the similarity between *Danafungia scruposa*_3509F and *Fungia fungites*_1207F is 99.81 %, which is greater than that of *Danafungia scruposa*_3509F and *Danafungia horrida*_0309F (98.37 %) (Supplementary Table 1).

**Table 1:** Information of samples, mitogenomes and sequence read archive (SRA).

Sample name	Sample location	Latitude/Longitude	Species	Mitogenome GenBank No.	Mitogenome length (bp)	SRA accession No.
3039F	Lingyang Reef	16°26′46″N/ 111°38′35″E	*Ctenactis* *crassa*	PQ875194	16,292	SRR34222871
0620F	Zhizhang Shoal	15°59′23″N/ 114°38′10″E	*Ctenactis* *echinata*	PQ571311	16,663	SRR34170476
1112F	Zhongbei Shoal	16°04′17″N/ 114°24′51″E	*Cycloseris* *costulata*	PQ571312	16,451	SRR34225146
3006F	Lingyang Reef	16°26′46″N/ 111°38′35″E	*Cycloseris* *costulata*	PQ875195	16,450	SRR34169429
3639F	Beidao Island	16°35′10″N/ 112°11′15″E	*Cycloseris* *fragilis*	PQ875196	16,522	SRR34222870
0309F	Haijiu Shoal	15°37′59″N/ 114°35′21″E	*Danafungia* *horrida*	PQ571313	17,332	SRR34225147
3001F	Lingyang Reef	16°26′46″N/ 111°38′35″E	*Danafungia* *horrida*	PQ875197	16,771	SRR34225149
1602F	Meixi Shoal	15°25′38″N/ 114°12′28″E	*Danafungia* *horrida*	PQ539056	16,577	SRR34216863
3023F	Lingyang Reef	16°26′46″N/ 111°38′35″E	*Danafungia* *horrida*	PQ875198	16,771	SRR34216822
3509F	Huaguang Reef	16°9′41″N/ 111°24′18″E	*Danafungia* *scruposa*	PQ875199	17,061	SRR34225148
1207F	Meibin Shoal	16°02′14″N/ 114°11′32″E	*Fungia* *fungites*	PQ571314	16,455	SRR34201160
1203F	Meibin Shoal	16°02′14″N/ 114°11′32″E	*Fungia* *fungites*	PQ571315	16,342	SRR34208142
1213F	Meibin Shoal	16°02′14″N/ 114°11′32″E	*Halomitra* *clavator*	PQ571316	16,551	SRR34216823
HGh35	Huaguang Reef	16°15′26″N/ 111°38′58″E	*Heliofungia* *actiniformis*	PQ571317	17,065	SRR34216862
0603F	Zhizhang Shoal	15°59′23″N/ 114°38′10″E	*Herpolitha* *limax*	PQ571318	16,619	SRR34201161
0614F	Zhizhang Shoal	15°59′23″N/ 114°38′10″E	*Lithophyllon* *concinna*	PQ571319	16,459	SRR34208135
0402F	Jimeng Shoal	15°42′09″N/ 114°41′05″E	*Lithophyllon* *scabra*	PQ571321	17,014	SRR34201159
gdg124	Qinglan	19°33′9″N/ 110°53′48″E	*Lithophyllon* *undulatum*	PQ571320	17,026	SRR34222869
0206F	Lexi Shoal	15°51′41″N/ 114°25′23″E	*Lobactis* *scutaria*	PQ571322	16,811	SRR34201158
0918F	Biwei Shoal	16°11′24″N/ 114°47′14″E	*Pleuractis* *gravis*	PQ571323	16,497	SRR34173838
3127F	Quanfu Island	16°35′1″N/ 111°41′17″E	*Pleuractis* *moluccensis*	PQ875200	16,854	SRR34216864
0311F	Haijiu Shoal	15°37′59″N/ 114°35′21″E	*Polyphyllia* *talpina*	PQ571324	17,108	SRR34173837
3209F	Yinyu Reef	16°32′2″N/ 111°39′36″E	*Sandalolitha* *robusta*	PQ875201	17,309	SRR34216865
0307F	Haijiu Shoal	15°37′59″N/ 114°35′21″E	*Sandalolitha* *robusta*	PQ571325	17,399	SRR34173836

**Fig. 1:**
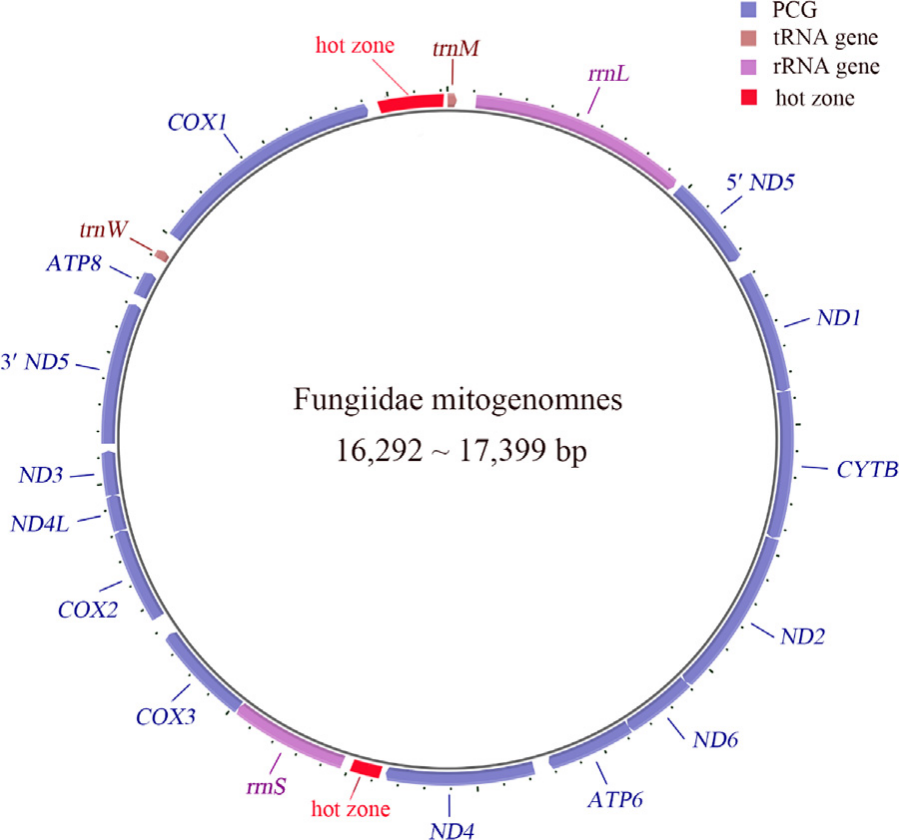
The maps of Fungiidae mitogenomes. The Fungiidae mitogenomes contain the same 13 PCGs (protein-coding genes) and two rRNA genes as other scleractinian corals. Comparing to the genes, the intergenic regions (IGR) are more diverse, especially in IGR*^COX1^*^-^*^trnM^* and IGR*^ND4^*^-^*^rrnS^* (the hot zones) of most Fungiidae mitogenomes, some minisatellite sequences were located. Primarily due to the existence of minisatellites in IGR*^COX1^*^-^*^trnM^*, the length of Fungiidae mitogenomes ranges from 16,292 to 17,399 bp.

The 24 Fungiidae mitogenomes all contain the same 13 PCGs (protein-coding genes) and two rRNA genes as other scleractinian corals (Fig. 1 and Supplementary Table 2). Comparing to the PCGs and rRNA genes, the intergenic regions (IGRs) are more diverse (Table 2). The nucleotide diversity of IGR*^COX1^*^-^*^trnM^* and IGR*^ND4^*^-^*^rrnS^* is highest (referred to as "hot zones") and in these two IGRs of most Fungiidae mitogenomes, minisatellite sequences were found (Table 3 and Supplementary material 1). The minisatellites located in IGR*^COX1^*^-^*^trnM^* have an average length of 277 bp, which is longer than those in IGR*^ND4^*^-^*^rrnS^* (average 214 bp). Furthermore, the length of IGR*^COX1^*^-^*^trnM^* minisatellites is strongly positively correlated with the length of mitogenomes (Pearson correlation coefficient 0.786), whereas the length of IGR*^ND4^*^-^*^rrnS^* minisatellites shows no significant correlation (Pearson correlation coefficient 0.092).

**Table 2:** The nucleotide diversity (Pi) of mitochondrial genes and IGRs.

Gene/IGR	Nucleotide diversity (Pi)
*ND4L*	0.0029
*ND1*	0.0037
*CYTB*	0.0040
*ND4*	0.0044
*rrnL*	0.0054
*ND2*	0.0055
*ATP6*	0.0060
*ND5*	0.0069
*COX3*	0.0076
COX2	0.0076
IGR (*COX3-COX2*)	0.0079
*ND6*	0.0079
*rrnS*	0.0080
IGR (3′*ND5-ND1*)	0.0117
*ND3*	0.0130
*ATP8*	0.0183
*COX1*	0.0309
IGR (*trnM-rrnL*)	0.0506
IGR (*ND4-rrnS*)	0.1329
IGR (*COX1*-*trnM*)	0.3425

*Note:* Nucleotide diversity was calculated for only five IGRs, other IGRs were excluded due to their short length (<100 bp), making them unsuitable for DNA barcode development.

**Table 3:** Information of minisatellites located in the IGRs.

Species	GenBank No.	IGR*^COX1^*^-^*^trnM^*			IGR*^ND4^*^-^*^rrnS^*		
		Minisatellite length (bp)	Minisatellite motif	Minisatellite position	Minisatellite length (bp)	Minisatellite motif	Minisatellite position
*Ctenactis* *crassa*	PQ875194	102	(GGCAATAATGTCGATAATAATTTGGATGGAG GGAATATTTGGATGCG)_2_	15,785–15,886	–	(None)^#^	–
*Ctenactis* *echinata*	PQ571311	483	(AATGTTAATGATAATTTTGAGATCGAATTTC CAGTTAATGAAGGGGGCCAG)_9_*	15,576–16,058	–	(None)	–
*Cycloseris* *costulata*	PQ571312	–	(None)^#^	–	–	(None)	–
*Cycloseris* *costulata*	PQ875195	–	(None)	–	–	(None)	–
*Cycloseris* *fragilis*	PQ875196	–	(None)	–	–	(None)	–
*Danafungia* *horrida*	PQ571313	595	(AGATACGGAGAAAGAGTGTGAGAAAGATTTA AGGACAAAAACATT)_8_* (GAAAAAGAGTGTGAAATAGATTTAATGAAAA AGAGTGTGAAATAGATTTAATGAAAAA GAGTGTGAAAAATATTTG)_3_*	16,585–17,179	194	(TTAAAAAGCCTTTGGTCTAAGTTAGTCTTTTA GTTTTGGGGATTTAAAAACTTTTGGTCTAAGTT AGACAGACGGGCCTGTCCTTTGGTTTTAAGGA)2*	8885–9078
*Danafungia* *horrida*	PQ875197	217	(CTAATAATTTAAATGAGGGGGTTCCAATGCC TGCCG)_6_*	16,092–16,308	484	(TTTGGTCTAAGTTAGTCTTTTAGTTTTGGGGA TTTAAAAACTTTTGGTCTAAGTTAGACAGGCG GGTCTGTCCTTTGGTTTTAAGGATTAAAAAGCT)5*	8896–9379
*Danafungia* *horrida*	PQ539056	217	(CTAATAATTTAAATGAGGGGGTTCCAATGCC TGCCG)_6_*	15,898–16,114	290	(TTTGGTCTAAGTTAGTCTTTTAGTTTTGGGGA TTTAAAAACTTTTGGTCTAAGTTAGACAGGCG GGTCTGTCCTTTGGTTTTAAGGATTAAAAAGCT)3*	8896–9185
*Danafungia* *horrida*	PQ875198	217	(CTAATAATTTAAATGAGGGGGTTCCAATGCC TGCCG)_6_*	16,092–16,308	484	(TTTGGTCTAAGTTAGTCTTTTAGTTTTGGGGA TTTAAAAACTTTTGGTCTAAGTTAGACAGGCG GGTCTGTCCTTTGGTTTTAAGGATTAAAAAGCT)5*	8896–9379
*Danafungia* *scruposa*	PQ875199	260	(TGATTAGACAAGGAGCAATAACATTGAGTTC TTTAAAAAAAAGTTGCTACCC)_5_*	16,790–17,049	387	(TAAGGATTAAAAAGCTTTTGGTCTAAGCTAGTCTTTTG GTTTCGGAGATTAGAAAACTTTTGGTTTAAGTTAGACA AGCTGATTAGTCTTTTGGTTT)4*	8960–9346
*Fungia* *fungites*	PQ571314	54	(CTGAAACTGTTAGTGCTGCAGCTTCCT)_2_*	16,204–16,257	291	(TAGTCTTTTAGTTTTGGGGATTAAAAAGCTTTTG ATCTAAGT)6*	8946–9236
*Fungia* *fungites*	PQ571315	153	(TCCAGTAAATCAGGAATTAAATAATGCAAAT CAGGGTGTTGACGGAGAACA)_3_*	15,692–15,844	110	(TCTAAGTTAGACAGGCTAGTTTGTCCTTTGGTTTTGG GGATTAAAAAGCTTTTGG)2	8994–9103
*Halomitra* *clavator*	PQ571316	108	(CTGAAACTGTTAGTGCTGCAGCTTCCT)_4_*	16,246–16,353	333	(TAGTCTTTTAGTTTTGGGGATTAAAAAGCTTTT GATCTAAGT)7*	8947–9279
*Heliofungia* *actiniformis*	PQ571317	335	(CTAAAACGGAATCCACTAATGCTGACGTACA AATTAGTGAAGATG)_5_*; (TTTGTATTTACTGGTGGGGGGTCTTTTGTTGA TGTTGTGTGATTATTTTTATATT)_2_	16,205–16,339; 16,479–16,588	74	(GTTAGTCTTTTGGTTTTGGGGATTAGAAA GCTGGCTG)2*	8954–9027
*Herpolitha* *limax*	PQ571318	180	(GAAAGTTGTAATCAAGAATCATCGGATGCTG GGGGGGAAATAAAT)_4_*	16,020–16,199	169	(CTTTTGGTCTAACTTAGACGACAGGCTGGTCTGTC CTTTGGTTTTGGGGATTAACCA)3*	8623–8791
*Lithophyllon* *concinna*	PQ571319	84	(CCCTGCCGCTAATAATGTTAATGAAGGGGTT CAGGTACCTGC)_2_	15,812–15,895	110	(CTTTTGGTCTAAGTTAGACAGGCGGGTCTGTCCTT TGGTTTTGGGGATTAAAAAG)2	8936–9045
*Lithophyllon* *scabra*	PQ571321	225	(AGAGTGTGAGAAAGATTTAAAGACAAAAA CATCAGATACGGAGAA)_2_(ATTTAATGAAAAA GAGTGTGAAATAG)_4_*	16,570–16,794	174	(GATTAAAAAGCTTTTGGTCTAAGTTA)5*	8884–9057
*Lithophyllon* *undulatum*	PQ571320	294	(AGAGTGTGAGAAAGATTTAAAGACAAAAA CATCAGATACGGAGAA)_2_(ATTTAATGAAAAA GAGTGTGAAATAG)_3_(GAAAAAGAGTGTGAAA AATATTTGGAAAAAGACCTGTTTAATGA)_2_	16,570–16,863	174	(GATTAAAAAGCTTTTGGTCTAAGTTA)5*	8884–9057
*Lithophyllon* *undulatum*	LC818211 ^✝^	225	(AGAGTGTGAGAAAGATTTAAAGACAAAAA CATCAGATACGGAGAA)_2_(ATTTAATGAAAAA GAGTGTGAAATAG)_3_*	16,570–16,794	174	(GATTAAAAAGCTTTTGGTCTAAGTTA)5*	8884–9057
*Lobactis* *scutaria*	PQ571322	152	(GTAAAAAAGCAGGTAGCCATCCTGGAAACA GGTAG)_2_(AAAAAGCAGGTAGCCATCCTGGAA ACAGGTAGGTAGCCATCCTGGAAACAGGTA GGT)2(AGGTAGCCATCCTGGAAACAGGT)_4_*	16,629–16,780	304	(TTTGGTCTAAGTTAGACACGCTGATTAGTCTTTTAGTT TTAAGGATTAAAAAGCG)4*; (AAAGCTTTTGGTCTAAGTTAGTCTTTTGGTTTCGGAGATTCG)2	8933–9152; 9244–9327
*Pleuractis* *gravis*	PQ571323	–	(None)	–	–	(None)	–
*Pleuractis* *moluccensis*	PQ875200	–	(None)	–	–	(None)	–
*Pleuractis* *paumotensis*	LC818212 ^✝^	–	(None)	–	–	(None)	–
*Polyphyllia* *talpina*	PQ571324	505	(GATTTACAAACCGAATTATTGAATATGGAGGG GGAAATTAATACCGATTTACAAACCGAATTATT GAATATGGAGGGGGAAGTTAATACC)_4_*(GAGTG GAAATTAATGAGGATTTACAAACCGAATTATTG AATATGGAGGGGGAAACTAATACCGATTTAA)_2_	16,373–16,877	110	(AGCTTTTGGTCTAAGTTAGACAGGCTGATCTGT CCTTTGGTTTTGGGGATTAAAA)2*	9237–9346
*Sandalolitha* *robusta*	PQ875201	448	(TTTGCTACTGAGGGAGAGGTTGATGAAAGT TTGGAAAAAGAGTTA)_3_*(GTTTAAAAAAAGA ATAATAATGATGACATGATAAATAAGGATTT TTGGTTTTGGG)_2_(TAAAAAAAGAATAATAAT GATGACATGATAAATAAAAAAAGAATAATA ATGATGACATAATAAA)_2_	16,745–17,192	104	(CTTTTGGTCTAAGTTAGACAGGTTGGTTAGTC TTTTAGTTTTGGGGATT)2*	8936–9039
*Sandalolitha* *robusta*	PQ571325	601	(AGTTTGGAAAAAGAGTTATTTGCTACTGAG GGAGAGGTTGATGAAAGTTTGGAAGAAGAA TTGTTTGCTACTGAGGGAGAGGTTGATGAA)_2_ (TTTGCTACTGAGGGAGAGGTTGATGAAAGT TTGGAAAAAGAGTTA)_2_*(GTTTAAAAAAAGAA TAATAATGATGACATGATAAATAAGGATTTTT GGTTTTGGG)_2_(TAAAAAAAGAATAATAATGAT GACATGATAAATAAAAAAAGAATAATAATG ATGACATAATAAA)_2_	16,682–17,282	104	(CTTTTGGTCTAAGTTAGACAGGTTGGTTAGTC TTTTAGTTTTGGGGATT)2*	8936–9039
*Sandalolitha* *robusta*	LC818214 ^✝^	315	(GGTTTGGAAAAAGAAGCATCCGATATTAACG AAGAAATTGACAAC)_2_; (TTTGCTACTGAGGGAGAGGTTGATGAAAGTTT GGAAAAAGAGTTA)_5_*	16,109–16,198; 16,757–16,981	104	(CTTTTGGTCTAAGTTAGACAGGTTGGTTAGTC TTTTAGTTTTGGGGATT)2*	8936–9039
*Podabacia* *crustacea*	LC818213 ^✝^	315	(GGTTTGGAAAAAGAAGCATCCGATATTAACG AAGAAATTGACAAC)_2_; (TTTGCTACTGAGGGAGAGGTTGATGAAAGTTT GGAAAAAGAGTTA)_5_*	16,098–16,187; 16,746–16,970	104	(CTTTTGGTCTAAGTTAGACAGGTTGGTTAGTC TTTTAGTTTTGGGGATT)2*	8936–9039

*Note:* * Imperfect minisatellite, which contains variations within its repeat pattern. ^#^ No minisatellite was found. ^✝^ Sequences retrieved from GenBank.

The same species (e.g. *Danafungia horrida* and *Sandalolitha robusta*) may contain the same minisatellite motifs, but the number of repeats of motifs can vary (Table 3).

**Fig. 2:**
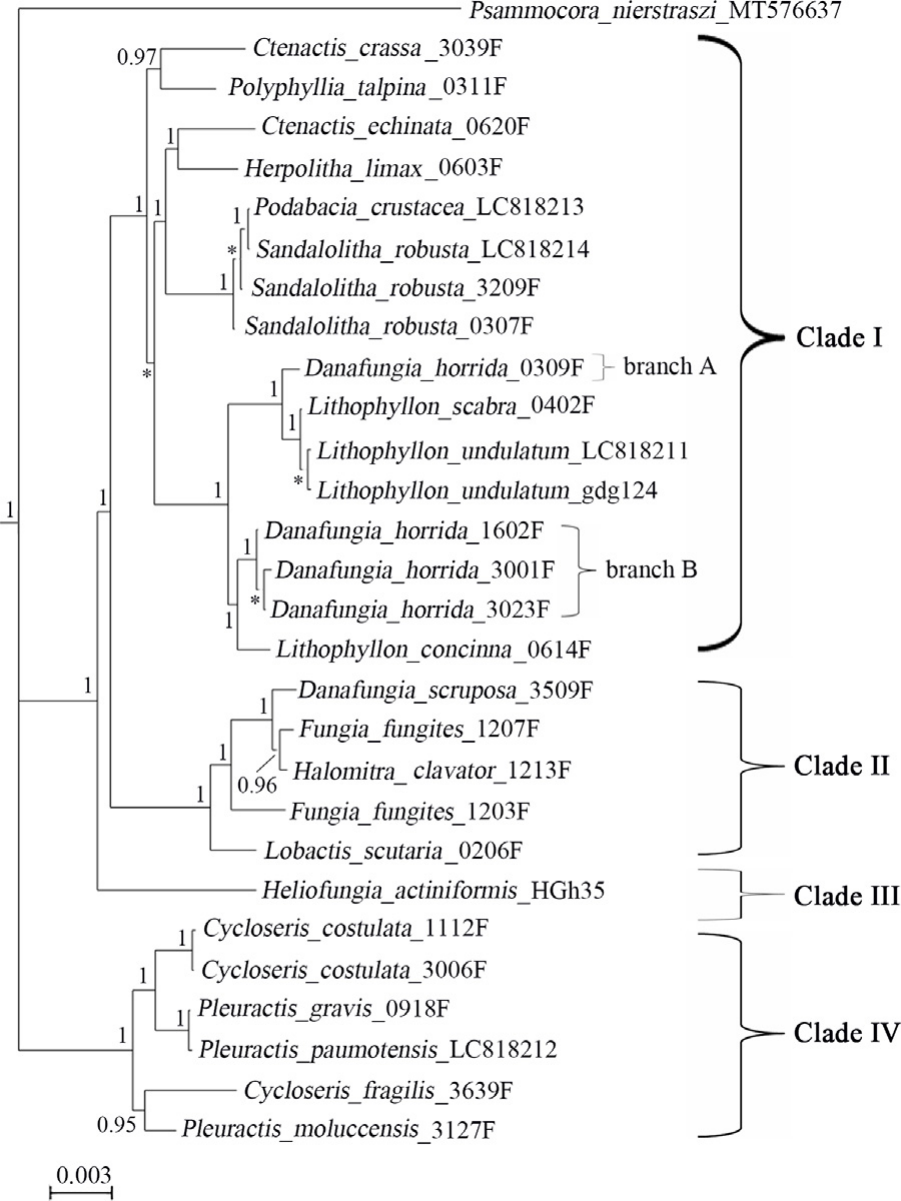
The Bayesian phylogenetic tree of Fungiidae based on 13 protein-coding genes. Numbers at nodes indicate Bayesian posterior probabilities. Stars indicate branch support values under 0.80.

The Bayesian phylogenetic trees of Fungiidae based on 13 protein-coding genes (PCGs) was constructed. Consistent with the tree inferred from COI [14], the tree based on 13 PCGs divides Fungiidae into four phylogenetic clades (Fig. 2). Most genera are polyphyletic. Notably, only the mitogenomes of clade IV (*Cycloseris* and *Pleuractis*) don’t contain the minisatellite sequence (Table 3). On the level of species, the two *Fungia fungites* individuals locates in different branches: *Fungia fungites*_1207F is the Clade A described by Oku et al. (2020) and *Fungia fungites*_1203F is Clade B [15]. Four *Danafungia horrida* individuals also locates in two different branches (Fig. 2). The three individuals of branch B share the same minisatellite motif, which differs from the motif found in branch A (Table 3).

**Fig. 3:**
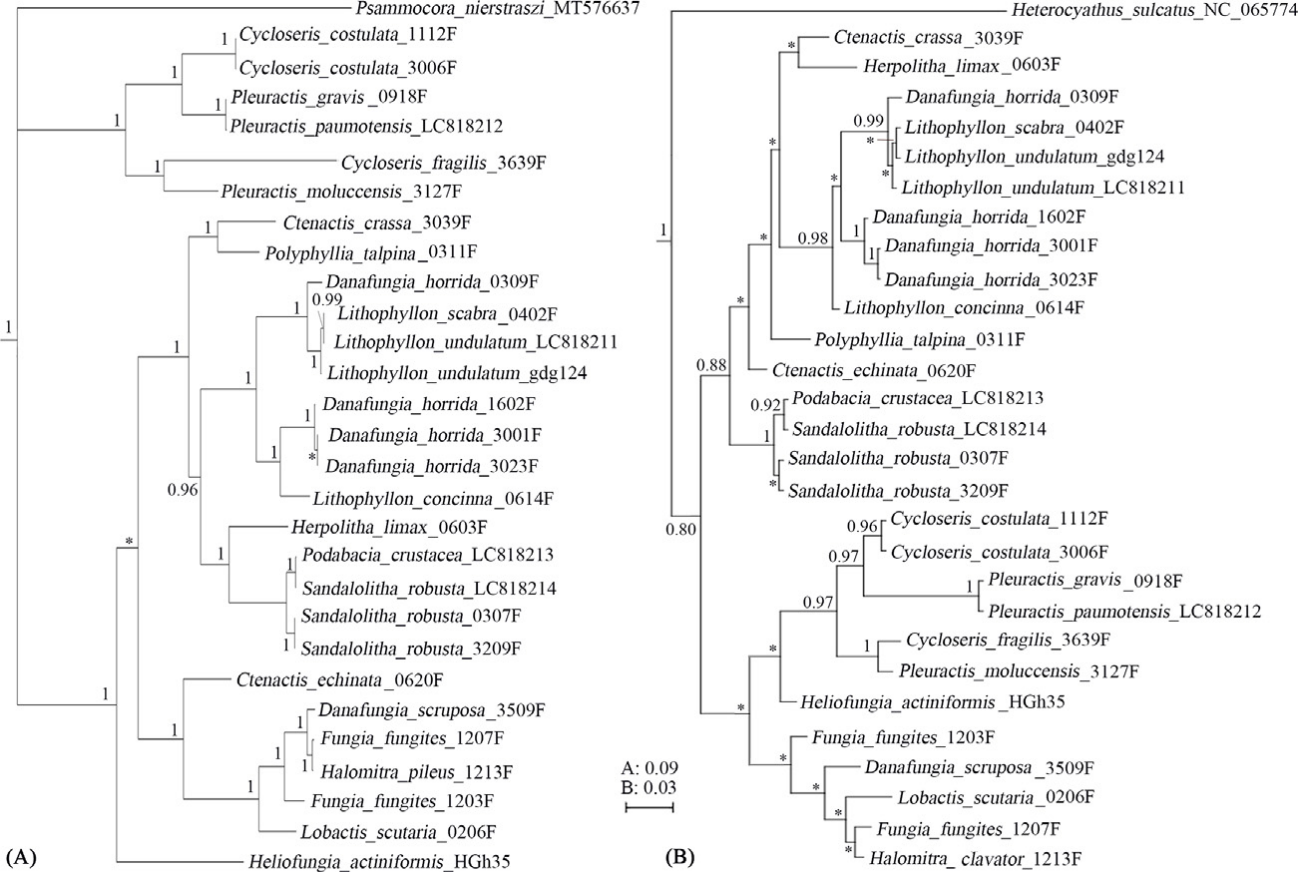
The Bayesian phylogenetic trees based on IGR*^COX1-trnM^* (A) and IGR*^ND4-rrnS^* (B). Numbers at nodes indicate Bayesian posterior probabilities. Stars indicate branch support values under 0.80. The scale bar of IGR*^COX1-trnM^* tree is 30 times longer than that of 13 PCGs tree.

The phylogenetic trees based on the two hot zones (IGR*^COX1^*^-^*^trnM^* and IGR*^ND4^*^-^*^rrnS^*) (Fig. 3) yield results similar to those of the 13-PCGs tree, but with higher resolution. The scale bars for the IGR*^COX1^*^-^*^trnM^* and IGR*^ND4^*^-^*^rrnS^* trees are 30 times and 10 times longer, respectively, than that of the 13-PCGs tree.

## 4 Experimental Design, Materials and Methods

### 4.1 Samples collection and identification

The Fungiidae samples were collected at Hainan fringe reefs, Xisha Islands and Zhongsha Atoll (all located in the South China Sea) from 2020 to 2024 (Table 1). The longitude and the latitude of sample location range from 110°53′48″ to 114°47′14″E, and from 15°25′38″N to 19°33′9"N, respectively. Coral sampling was conducted under permits approved by the Department of Ocean and Fisheries of Hainan Province (China). Morphological species identification was based on colony shape, polyp structure, and septa [1,2], while molecular identification focused on a portion of the cytochrome oxidase I (COI, 500 bp) gene using fungiid-specific primers FungCOIfor1 5′-CTGCTCTTAGTATGCTTGTA-3′ and FungCOIrev2 5′-TTGCACCCGCTAATACAG-3′ [5], as well as an approximately 800 bp region of rDNA using the primers ITS4 5′-TCCTCCGCTTATTGATATGC-3′ [6] and A18S 5′-GATCGAACGGTTTAGTGAGG-3′ [7].

A total of 18 species from 12 genera were identified (Table 1). For samples that were difficult to identify or prone to confusion, e.g. *Danafungia horrida, Fungia fungites, Cycloseris costulata* and *Sandalolitha robusta*, we selected two or more samples for mitogenome sequencing. In total, 24 samples were selected (Table 1).

### 4.2 DNA sequencing

Genomic DNA was extracted using the Tianamp Marine Animals DNA Kit (TIANGEN, CHINA). After DNA isolation, 1 μg of purified DNA was fragmented to ∼500 bp using the Covaris M220 system. The short-insert libraries were constructed according to the manufacturer’s instructions (TruSeq™ Nano DNA Sample Prep Kit, Illumina) and then the sequencing was performed on an Illumina NovaSeq 6000 platform (BIOZERON Co., Ltd, Shanghai, China) with 150 bp paired-end reads length. Raw Illumina sequencing reads have been deposited in the NCBI Sequence Read Archive (SRA), and SRA accession numbers were assigned (Table 1).

### 4.3 Mitogenome assembly and gene annotation

Prior to assembly, raw reads were filtered by Trimmomatic 0.39 [16] to remove the reads with adaptors, the reads showing a quality score below 20 (*Q* < 20), the reads containing a percentage of uncalled bases (“N” characters) ≥ 10 % and the duplicated sequences. The filtered reads were assembled into contigs using MitoZ v2.3, which typically produced multiple contigs. Manual curations were conducted for each dataset. Specifically, mitochondrial contigs with >80 % query coverage were identified through BLAST v2.8.1+ alignment against reference mitogenomes. These contigs were then manually ordered, oriented, and joined based on the structure of the corresponding reference mitogenomes. MUMmer 3.23 was subsequently used to confirm the circularity of the assembled mitogenomes. As a result of this process, each final mitochondrial genome assembly consisted of a single circular contig.

The assembled Fungiidae mitogenome sequences were imported into MITOS web server (http://mitos.bioinf.uni-leipzig.de/) [17] for gene annotation. The start and stop codon positions of each gene were manually corrected. Mitogenome visualization was performed using the Proksee online platform (https://proksee.ca/) [18].

### 4.4 Mitogenome analysis

The 24 Fungiidae mitogenomes sequenced in this study (Table 1), along with four additional Fungiidae mitogenomes retrieved from GenBank [12], were used for the following analysis.

#### 4.4.1 Mitogenome similarity, nucleotide diversity and minisatellite screening

The similarity among Fungiidae mitogenomes was calculated using the BLAST program of NCBI (https://blast.ncbi.nlm.nih.gov/). The nucleotide diversity (Pi) of protein coding genes, rRNA genes and IGRs was calculated using DnaSP version 5.0 [19]. The minisatellite sequences were screened with MISA software [20] and the imperfect minisatellites, which contains variations within its repeat pattern, were checked manually. The correlation between the length of minisatellites and the length of mitogenomes was calculated using the PEARSON function (for Pearson’s correlation coefficient) in Microsoft Excel.

#### 4.4.2 Phylogenetic analysis

Bayesian inference phylogeny trees were constructed based on 13 PCGs and the two hot zones (IGR*^COX1^*^-^*^trnM^* and IGR*^ND4^*^-^*^rrnS^*). For the 13-PCGs tree and IGR*^COX1^*^-^*^trnM^* tree, the *Psammocora nierstraszi* mitogenome (GenBank no MT576637) was set as outgroup, and for the IGR*^ND4^*^-^*^rrnS^* tree, *Heterocyathus sulcatus* mitogenome (NC_065774) was used as outgroup. Sequences were firstly aligned separately using MAFFT v.7.407 [21], and then Gblocks v.0.91b [22] was applied to remove ambiguously aligned regions using default settings. Based on the corrected Akaike Information Criterion (AICc) [23], the best partition scheme, as well as the best-fit nucleotide substitution models for the respective partitions, were detected by PartitionFinder 2 [24]. MrBayes v.3.2.7 [25] was used for the Bayesian inference analysis, and ETE Tree Browser [26] was used to visualize the resulting phylogeny tree.

## Limitations

Not applicable.

## Ethics Statement

The authors have read and followed the ethical requirements for publication in Data in Brief. The authors confirm the current work does not involve human subjects, animal experiments, or any data collected from social media platforms.

## CRediT authorship contribution statement

**Liwei Li:** Investigation. **Zhangwang Lu:** Methodology. **Zhiwei Liu:** Investigation. **Cheng Liang:** Investigation. **Jun Wang:** Data curation. **Yan Wang:** Conceptualization, Writing – review & editing.

## Data Availability

NCBIPQ571325 (Original data). NCBIPQ571324 (Original data). NCBIPQ571323 (Original data). NCBIPQ571322 (Original data). NCBIPQ875197 (Original data). NCBIPQ875196 (Original data). NCBIPQ875195 (Original data). NCBIPQ875194 (Original data). NCBIPQ571317 (Original data). NCBIPQ571316 (Original data). NCBIPQ571315 (Original data). NCBIPQ571314 (Original data). NCBIPQ571321 (Original data). NCBIPQ571320 (Original data). NCBIPQ571319 (Original data). NCBIPQ571318 (Original data). NCBIPQ571313 (Original data). NCBIPQ571312 (Original data). NCBIPQ571311 (Original data). NCBIPQ539056 (Original data). NCBIPQ875201 (Original data). NCBIPQ875200 (Original data). NCBIPQ875199 (Original data). NCBIPQ875198 (Original data).

## Appendix Supplementary materials

Image, application 1

Image, application 2

Image, application 3
