# Supplementary material for: Dataset of the complete mitogenomes of the mushroom corals Fungiidae
Source: Data Brief. 2025 Jul 8;61:111857. doi: 10.1016/j.dib.2025.111857 (PMC12311936; doi:10.1016/j.dib.2025.111857)
Supplement: Supplementary file 1 [file mmc1.docx]

**The minisatellite sequences located in the IGR (*COX1*-*trnM*) of Fungiidae mitogenomes. Some are imperfect minisatellite, which contains variations (marked in green) within its repeat pattern.**

>Ctenactis crassa_3039F

GGCAATAATGTCGATAATAATTTGGATGGAGGGAATAATGATTTGGATGCGGGCAATAATGTCGATAATAATTTGGATGGAGGGAATAATGATTTGGATGCG

>Ctenactis echinata_0620F

AATGTTAATGATAATTTTGAGATCGAATTTCCAGTTAATGAAGGGGGCCAGAATGTTAATGATAATTTTGAGATCGAATTTCCAGTTAATGAAGGGGGCCAGAATGTTAATGTTAATGATAATTTTGAGATCGAATTTCCAGTTAATGAAGGGGGCCAGAATGTTAATGTTAATGATAATTTTGAGATCGGATTTCCAGTTAATGAAGGGGGCCAGAATGTTAATGATAATTTTGAGATCGAGTTTCCAGTTAATGAAGGGGGCCAGAATGTTAATGTTAATGATAATTTTGAGATCGGATTTCCAGTTAATGAAGGGGGCCAGAATGTTAATGATAATTTTGAGATCGAGTTTCCAGTTAATGAAGGGGGCCAGAATGTTAATGTTAATGATAATTTTGAGATCGGATTTCCAGTTAATGAAGGGGGCCAGAATGTTAATGATAATTTTGAGATCGAATTTCCAGTTAATGAAGGGGGCCAG

>Danafungia horrida_0309F

AGATACGGAGAAAGAGTGTGAGAAAGATTTAAGGACAAAAACATTAGATACGGAGAAAGAGTGTGAGAAAGATTTAAGGACAAAAACATTAGATACGGAGAAAGAGTGTGAGAAAGATTTAAGGACAAAAACATTAGATACGGAGAAAGAGTGTGAGAAAGATTTAAGGACAAAAACATCAGATACGGAGAAAGAGTGTGAGAAAGATTTAAAGACAAAAACATCAGATACGGAGAAAGAGTGTGAGAAAGATTTAAAGACAAAAACATCAGATACGGAGAAAGAGTGTGAGAAAGATTTAAAGACAAAAACATCAGATACGGAGAAAAAGTGTGAAAAAGATTTGAAAAAAAAAACCATTTAATGAAAAAGAGTGTGAAATAGATTTAATGAAAAAGAGTGTGAAATAGATTTAATGAAAAAGAGTGTGAAATAGATTTAATGAAAAAGAGTGTGAAATAGATTTAATGAAAAAGAGTGTGAAATAGATTTAATGAAAAAGAGTGTGAAAAATATTTGGAAAAAGAGTGTGAAATAGATTTAATGAAAAAGAGTGTGAAATAGATTTAATGAAAAAGAGTGTGAAAAATATTTG

>Danafungia horrida_3001F

CTAATAATTTAAATGAGGGGGTTCCAATGCCTGCCGCTAATAATTTAAATGAGGGGGTTCCAATGCCTGCCGCTAATAATTTAAATGAGGGGGTTCCAATGCCTGCCGCTAATAATTTAAATGAGGGGGTTCCAATACCTGCCGATAATAATTTAAATGAGGGGGTTCCAATGCCTGCCGCTAATAATTTAAATGAGGGGGTTCCAATACCTGCCG

>Danafungia horrida_1602F

CTAATAATTTAAATGAGGGGGTTCCAATGCCTGCCGCTAATAATTTAAATGAGGGGGTTCCAATGCCTGCCGCTAATAATTTAAATGAGGGGGTTCCAATGCCTGCCGCTAATAATTTAAATGAGGGGGTTCCAATACCTGCCGATAATAATTTAAATGAGGGGGTTCCAATGCCTGCCGCTAATAATTTAAATGAGGGGGTTCCAATACCTGCCG

>Danafungia horrida_3023F

CTAATAATTTAAATGAGGGGGTTCCAATGCCTGCCGCTAATAATTTAAATGAGGGGGTTCCAATGCCTGCCGCTAATAATTTAAATGAGGGGGTTCCAATGCCTGCCGCTAATAATTTAAATGAGGGGGTTCCAATACCTGCCGATAATAATTTAAATGAGGGGGTTCCAATGCCTGCCGCTAATAATTTAAATGAGGGGGTTCCAATACCTGCCG

>Danafungia scruposa_3509F

TGATTAGACAAGGAGCAATAACATTGAGTTCTTTAAAAAAAAGTTGCTACCCTGATTAGACAAGGAGCAATAACATTGAGTTCTTTAAAAAAAAGTTGCCACCTTGATTAGACAAGGAGCAATAACATTGAGTTCTTTAAAAAAAAGTTGCTACCCTGATTAGACAAGGAGCAATAACATTGAGTTCTTTAAAAAAAAGTTGCCACCTTGATTAGACAAGGAGCAATAACATTGAGTTTTTTAAAAAAAAGTTGCCACCC

>Fungia fungites_1207F

CTGAAACTGTTAGTGCTGCAGCTTCCTCTGAAACTGTTAGTGCTGCAATTTCCC

>Fungia fungites_1203F

TCCAGTAAATCAGGAATTAAATAATGCAAATCAGGGTGTTGACGGAGAACATCCAGTAAATCAGGAATTAAATAATGCAAATCAGGGTGTTGACGGAGAACATCCAGTAAATCAGGAATTAAATAATGCAAATCAGGGTGTTGATGGAGAACC

>Halomitra pileus_1213F

CTGAAACTGTTAGTGCTGCAGCTTCCTCTGAAACTGTTAGTGCTGCAGCTTCCTCTGAAACTGTTAGTGCTGCAGCTTCCT CTGAAACTGTTAGTGCTGCAATTTCCC

>Heliofungia actiniformis_HGh35

CTAAAATGGAATCCACTAATATTGACGTACAAATTAGTGATGATGCTAAAACGGAATCCACTAATGCTGACGTACAAATTAGTGAGGACTCTAAAACGGAATCCACTAATGCTGACGTACAAATTAGTGAAGATGCTAAAACGGAATCCACTAATGCTGACGTACAAATTAGTGAAGATGCTAAAACGGAATCCACTAATGCTGACGTACAAATTAGTGAAGCCT//TTTGTATTTACTGGTGGGGGGTCTTTTGTTGATGTTGTGTGATTATTTTTATATTTTTGTATTTACTGGTGGGGGGTCTTTTGTTGATGTTGTGTGATTATTTTTATATT

>Herpolitha limax_0603F

GAAAGTTGTAATCAAGAATCATCGGATGCTGGGGGGGAAATAAATGAAAGTTGTAATAAAGAATCATCGGATACTGGGGGGGAAATAAATGAAAGTTGTAATCAAGAACCATCGGATACTGGGGAGGAAATTAATGAAAGTTGTGATAAAGAATCATCGGATACTGGGGAGGAAATAAAT

>Lithophyllon concinna_0614F

CCCTGCCGCTAATAATGTTAATGAAGGGGTTCAGGTACCTGCCCCTGCCGCTAATAATGTTAATGAAGGGGTTCAGGTACCTGC

>Lithophyllon scabra_0402F

AGAGTGTGAGAAAGATTTAAAGACAAAAACATCAGATACGGAGAAAGAGTGTGAGAAAGATTTAAAGACAAAAACATCAGATACGGAGAAAAAGTGTGAAAAAGATTTGAAAAAAAAAACCATTTAATGAAAAAGAGTGTGAAATAGATTTAATGAAAAAGAGTGTGAAATAGATTTAATGAAAAAGAGTGTGAAATAGATTTAAGGAAAAAGAGTGTGAAAAAT

>Lithophyllon undulatum_gdg124

AGAGTGTGAGAAAGATTTAAAGACAAAAACATCAGATACGGAGAAAGAGTGTGAGAAAGATTTAAAGACAAAAACATCAGATACGGAGAAAAAGTGTGAAAAAGATTTGAAAAAAAAAACCATTTAATGAAAAAGAGTGTGAAATAGATTTAATGAAAAAGAGTGTGAAATAGATTTAATGAAAAAGAGTGTGAAATAGATTTAAGGAAAAAGAGTGTGAAAAATATTTGGAAAAAGACCTGTTTAATGAGAAAAAGAGTGTGAAAAATATTTGGAAAAAGACCTGTTTAATGA

>Lithophyllon undulatum_LC818211

AGAGTGTGAGAAAGATTTAAAGACAAAAACATCAGATACGGAGAAAGAGTGTGAGAAAGATTTAAAGACAAAAACATCAGATACGGAGAAAAAGTGTGAAAAAGATTTGAAAAAAAAAACCATTTAATGAAAAAGAGTGTGAAATAGATTTAATGAAAAAGAGTGTGAAATAGATTTAATGAAAAAGAGTGTGAAATAGATTTAAGGAAAAAGAGTGTGAAAAAT

>Lobactis scutaria_0206F

GTAAAAAAGCAGGTAGCCATCCTGGAAACAGGTAGGTAAAAAAGCAGGTAGCCATCCTGGAAACAGGTAGGTAGCCATCCTGGAAACAGGTAGGTAAAAAGCAGGTAGCCATCCTGGAAACAGGTAGGTAGCCATCCTGGAAACAGGTAGGT

>Polyphyllia talpina_0311F

GATTTACAAACCGAATTATTGAATATGGAGGGGGAAATTAATACCGATTTACAAACCGAATTATTGAATATGGAGGGGGAAGTTAATACCGATTTACAAACCGAATTATTGAATATGGAGGGGGAAATTAATACCGATTTACAAACCGAATTATTGAATATGGAGGGGGAAGTTAATACCGATTTACAAACCGAATTATTGAATATGGAGGGGGAAATTAATACCGATTTACAAACCGAATTATTGAATATGGAGGGGGAAGTTAATACCGATTTACAAACCGAATTATTGAATATGGAGGGGGAAACTAATGAGGATTTACAAACCGAATTATTGAATATGGAGGGGGAAACTAATACCGATTTAGGAGTGGAAATTAATGAGGATTTACAAACCGAATTATTGAATATGGAGGGGGAAACTAATACCGATTTAAGAGTGGAAATTAATGAGGATTTACAAACCGAATTATTGAATATGGAGGGGGAAACTAATACCGATTTAA

>Sandalolitha robusta_3209F

TTTGCTACTGAGGGAGAGGTTGATGAAAGTTTGGAAAAAGAGTTATTTGCTACTGAGGGAGAGGTTGATGAAAGTTTGGAAAAAGAGTTATTTGCTACTGAGGGAGGGGTTGATGAAAGTTTGGAAAAAGAGCCATCTAATGAAGAAGAGGGTAGGCCAAGAAAAAGACCACGATTACATAATAAATAATAATGTTTGATTTCTGGAGTTTAAAAAAAGAATAATAATGATGACATGATAAATAAGGATTTTTGGTTTTGGGGTTTAAAAAAAGAATAATAATGATGACATGATAAATAAGGATTTTTGGTTTTGGGGTTTAAAAAAAGAATAATAATGATGACATGATAAATAAAAAAAGAATAATAATGATGACATAATAAATAAAAAAAGAATAATAATGATGACATGATAAATAAAAAAAGAATAATAATGATGACATAATAAA

>Sandalolitha robusta_0307F

AGTTTGGAAAAAGAGTTATTTGCTACTGAGGGAGAGGTTGATGAAAGTTTGGAAGAAGAATTGTTTGCTACTGAGGGAGAGGTTGATGAAAGTTTGGAAAAAGAGTTATTTGCTACTGAGGGAGAGGTTGATGAAAGTTTGGAAGAAGAATTGTTTGCTACTGAGGGAGAGGTTGATGAAAGTTTGGAAAAAGAGTTATTTGCTACTGAGGGAGAGGTTGATGAAAGTTTGGAAAAAGAGTTATTTGCTACTGAGGGAGGGGTTGATGAAAGTTTGGAAAAAGAGCCATCTAATGAAGAAGAGGGTAGGCCAAGAAAAAGACCACGATTACATAATAAATAATAATGTTTGATTTCTGGAGTTTAAAAAAAGAATAATAATGATGACATGATAAATAAGGATTTTTGGTTTTGGGGTTTAAAAAAAGAATAATAATGATGACATGATAAATAAGGATTTTTGGTTTTGGGGTTTAAAAAAAGAATAATAATGATGACATGATAAATAAAAAAAGAATAATAATGATGACATAATAAATAAAAAAAGAATAATAATGATGACATGATAAATAAAAAAAGAATAATAATGATGACATAATAAA

>Sandalolitha robusta_LC818214

GGTTTGGAAAAAGAAGCATCCGATATTAACGAAGAAATTGACAACGGTTTGGAAAAAGAAGCATCCGATATTAACGAAGAAATTGACAAC//TTTGCTACTGAGGGAGAGGTTGATGAAAGTTTGGAAGAAGAATTGTTTGCTACTGAGGGAGAGGTTGATGAAAGTTTGGAAGAAGAATTGTTTGCTACTGAGGGAGAGGTTGATGAAAGTTTGGAAAAAGAGTTATTTGCTACTGAGGGAGAGGTTGATGAAAGTTTGGAAAAAGAGTTATTTGCTACTGAGGGAGGGGTTGATGAAAGTTTGGAAAAAGAGCCA

>Podabacia crustacea_LC818213

GGTTTGGAAAAAGAAGCATCCGATATTAACGAAGAAATTGACAACGGTTTGGAAAAAGAAGCATCCGATATTAACGAAGAAATTGACAAC//TTTGCTACTGAGGGAGAGGTTGATGAAAGTTTGGAAGAAGAATTGTTTGCTACTGAGGGAGAGGTTGATGAAAGTTTGGAAGAAGAATTGTTTGCTACTGAGGGAGAGGTTGATGAAAGTTTGGAAAAAGAGTTATTTGCTACTGAGGGAGAGGTTGATGAAAGTTTGGAAAAAGAGTTATTTGCTACTGAGGGAGGGGTTGATGAAAGTTTGGAAAAAGAGCCA

**The minisatellite sequences located in the IGR (*ND4-rrnS*) of Fungiidae mitogenomes.**

>Danafungia_horrida_0309F

TTAAAAAGCCTTTGGTCTAAGTTAGTCTTTTAGTTTTGGGGATTTAAAAACTTTTGGTCTAAGTTAGACAGACGGGCCTGTCCTTTGGTTTTAAGGATTAAAAAGCCTTTGGTCTAAGTTAGTCTTTTAGTTTTGGGGATTTAAAAACTTTTGGTCTAAGTTAGACAGGCGGGTCTGTCCTTTGGTTTTGAGGA

>Danafungia horrida_3001F

TTTGGTCTAAGTTAGTCTTTTAGTTTTGGGGATTTAAAAACTTTTGGTCTAAGTTAGACAGGCGGGTCTGTCCTTTGGTTTTAAGGATTAAAAAGCTTTTGGTCTAAGTTAGTCTTTTAGTTTTGGGGATTTAAAAACTTTTGGTCTAAGTTAGACAGGCGGGTCTGTCCTTTGGTTTTAAGGATTAAAAAGCTTTTGGTCTAAGTTAGTCTTTTAGTTTTGGGGATTTAAAAACTTTTGGTCTAAGTTAGACAGGCGGGTCTGTCCTTTGGTTTTAAGGATTAAAAAGCTTTTGGTCTAAGTTAGTCTTTTAGTTTTGGGGATTTAAAAACTTTGGATCTAAGTTAGACAGGCGGGTCTGTCCTTTGGTTTTGGGGATTAGCCAGCCTTTGGTCTAAGTTAGTCTTTTAGTTTTGG-GATTAAAAAGCTTTTGGTCTAAGCTAGACAGGCGGG

TCTGTCCTTTGGTTTTGGGGATTAAAAAGCT

>Danafungia horrida_1602F

TTTGGTCTAAGTTAGTCTTTTAGTTTTGGGGATTTAAAAACTTTTGGTCTAAGTTAGACAGGCGGGTCTGTCCTTTGGTTTTAAGGATTAAAAAGCTTTTGGTCTAAGTTAGTCTTTTAGTTTTGGGGATTTAAAAACTTTGGATCTAAGTTAGACAGGCGGGTCTGTCCTTTGGTTTTGGGGATTAGCCAGCCTTTGGTCTAAGTTAGTCTTTTAGTTTTGG-GATTAAAAAGCTTTTGGTCTAAGCTAGACAGGCGGG

TCTGTCCTTTGGTTTTGGGGATTAAAAAGCT

>Danafungia horrida_3023F

TTTGGTCTAAGTTAGTCTTTTAGTTTTGGGGATTTAAAAACTTTTGGTCTAAGTTAGACAGGCGGGTCTGTCCTTTGGTTTTAAGGATTAAAAAGCTTTTGGTCTAAGTTAGTCTTTTAGTTTTGGGGATTTAAAAACTTTTGGTCTAAGTTAGACAGGCGGGTCTGTCCTTTGGTTTTAAGGATTAAAAAGCTTTTGGTCTAAGTTAGTCTTTTAGTTTTGGGGATTTAAAAACTTTTGGTCTAAGTTAGACAGGCGGGTCTGTCCTTTGGTTTTAAGGATTAAAAAGCTTTTGGTCTAAGTTAGTCTTTTAGTTTTGGGGATTTAAAAACTTTGGATCTAAGTTAGACAGGCGGGTCTGTCCTTTGGTTTTGGGGATTAGCCAGCCTTTGGTCTAAGTTAGTCTTTTAGTTTTGG-GATTAAAAAGCTTTTGGTCTAAGCTAGACAGGCGGG

TCTGTCCTTTGGTTTTGGGGATTAAAAAGCT

>Danafungia scruposa_3509F

TAAGGATTAAAAAGCTTTTGGTCTAAGCTAGTCTTTTGGTTTCGGAGATTAGAAAACTTTTGGTTTAAGTTAGACAAGCTGATTAGTCTTTTGGTTTTAAGGATTAAAAAGCTTTTGGTCTAAGCTAGTCTTTTGGTTTCGGAGATTAGAAAACTTTTGGTTTAAGTTAGACAAGCTGATTAGTCTTTTGGTTTTAAGGATTAAAAAGCTTTTGGTCTAAGCTAGTCTTTTGGTTTCGGAGATTAGAAAACTTTTGGTTTAAGTTAGACAAGCTGATTAGTCTTTTGGTTTTAAGGATTAAAAAGCTTTTGGTCTAAGCTAGTCTTTTGGTTTCGGAGATTAGAAAGCTTTTGGTCTAAGTTAGACAAGCTGATTAGTCTTTTAGTTT

>Fungia_fungites_1207F

TAGTCTTTTAGTTTTGGGGATTAAAAAGCTTTTGATCTAAGTtagacaggctagtTTGTCCTTTGGTTTTGGGGATTAAAAAGCTTTTGGTCTAAGTTAGTCTTTTGGTTTCGGAGATTAGAAAACTTTTGGTTTAAGTtagacaagctgatTAGTCTTTTGGTTTTGGGGATTAAAAAGCTTTTGGTCTAAGTTAGTCTTTTGGTTTCGGAGATTAGAAAGCTTTTGGTCTAAGTtagacaagctggtTAGTCTTTTAGTTTTGGGGATTAAAAAGCTTTTGATCTAAGT

> Fungia_fungites_1203F

TCTAAGTTAGACAGGCTAGTTTGTCCTTTGGTTTTGGGGATTAAAAAGCTTTTGGTCTAAGTTAGACAGGCTAGTTTGTCCTTTGGTTTTGGGGATTAAAAAGCTTTTGG

>Halomitra_clavator_1213F

TAGTCTTTTAGTTCTAAGGATTAAAAAGCTTTTGGTCTAAGTTAGTCTTTTGGTTTCGGAGATTAGAAAACTTTTGGTTTAAGTTAGACAAGCTGATTAGTCTTTTGGTTTTAAGGATTAAAAAGCTTTTGGTCTAAGTTAGTCTTTTGGTTTCGGAGATTAGAAAACTTTTGGTTTAAGTTAGACAAGCTGATTAGTCTTTTGGTTTTGGGGATTAAAAAGCTTTTGGTCTAAGTTAGTCTTTTGGTTTCGGAGATTAGAAAGCTTTTGGTCTAAGTTAGACAAGCTGGTTAGTCTTTTAGTTTTGGGGATTAAAAAGCTTTTGATCTAAGT

>Heliofungia_actiniformis_HGh35

GTTAGTCTTTTGGTTTTGGGGATTAGAAAGCTGGCTGGTTAGTCTTTTAGTTTTGGGGATTAAAAAGCTGGTCT

>Herpolitha_limax_0603F

CTTTTGGTCTAACTTAGACGACAGGCTGGTCTGTCCTTTGGTTTTGGGGATTAACCACTTTTGGTCTAAGTTAGACAG---GCTGGTCTGTCCTTTGGTTTTGGGGATTAGCCAgCTTTTGGTCTAACCTAGACGACAGGCTGGTCTGTCCTTTGGTTTTGGGGACTAACCA

>Lithophyllon concinna_0614F

CTTTTGGTCTAAGTTAGACAGGCGGGTCTGTCCTTTGGTTTTGGGGATTAAAAAGCTTTTGGTCTAAGTTAGACAGGCGGGTCTGTCCTTTGGTTTTGGGGATTAAAAAG

>Lithophyllon_scabra_0402F

GATTAAAAAGCCTTTGGTCTAAGTTAgtcttttagttttgggGATTTAAAAACTTTTGGTCTAAGTTAgacaggcgggtctgtcctttggttttgagGATTAAAAAGCTTTTGGTCTAAGTTAGATTAAAAAACTTTTGATCTAAGTTAGACCAAAAGCTTTTGGTCTAAACGA

>Lithophyllon_undulatum_gdg124

GATTAAAAAGCCTTTGGTCTAAGTTAgtcttttagttttgggGATTTAAAAACTTTTGGTCTAAGTTAgacaggcgggtctgtcctttggttttgagGATTAAAAAGCTTTTGGTCTAAGTTAGATTAAAAAACTTTTGATCTAAGTTAGACCAAAAGCTTTTGGTCTAAACGA

>Lithophyllon_undulatum_LC818211

GATTAAAAAGCCTTTGGTCTAAGTTAgtcttttagttttgggGATTTAAAAACTTTTGGTCTAAGTTAgacaggcgggtctgtcctttggttttgagGATTAAAAAGCTTTTGGTCTAAGTTAGATTAAAAAACTTTTGATCTAAGTTAGACCAAAAGCTTTTGGTCTAAACGA

>Lobactis scutaria_0206F

TTTGGTCTAAGTTAGACACGCTGATTAGTCTTTTAGTTTTAAGGATTAAAAAGCGTTTGGTCTAAGTTAGACACGCTGATTAGTCTTTTAGTTTTAAGGATTAAAAAGCGTTTGGTCTAAGTTAGATACGCTGATTAGTCTTTTAGTTTTAAGGATTAAAAAGCGTTTGGTCTAAGTTAGACACGCTGATTAGTCTTTTAGTTTTAAGGATTAAAAAGCG//AAAGCTTTTGGTCTAAGTTAGTCTTTTGGTTTCGGAGATTCGAAAGCTTTTGGTCTAAGTTAGTCTTTTGGTTTCGGAGATTCG

>Polyphyllia_talpina_0311F

AGCTTTTGGTCTAAGTTAGACAGGCTGATCTGTCCTTTGGTTTTGGGGATTAAAAAGCTTTTGGTCTAAGTTATACAGGCTGATCTGTCCTTTGGTTTTGGGGATTAAAA

>Sandalolitha_robusta_3209F

CTTTTGGTCTAAGTTAGACAGGTTGGTTAGTCTTTTAGTTTTGGGGATTtaaaaaCTTTTGGTCTAAGTTAAACAGGCTGGTCTGTCCTTTGGTTTTGGGGATT

>Sandalolitha_robusta_0307F

CTTTTGGTCTAAGTTAGACAGGTTGGTTAGTCTTTTAGTTTTGGGGATTtaaaaaCTTTTGGTCTAAGTTAAACAGGCTGGTCTGTCCTTTGGTTTTGGGGATT

>Sandalolitha_robusta_LC818214

CTTTTGGTCTAAGTTAGACAGGTTGGTTAGTCTTTTAGTTTTGGGGATTtaaaaaCTTTTGGTCTAAGTTAAACAGGCTGGTCTGTCCTTTGGTTTTGGGGATT

>Podabacia_crustacea_LC818213

CTTTTGGTCTAAGTTAGACAGGTTGGTTAGTCTTTTAGTTTTGGGGATTtaaaaaCTTTTGGTCTAAGTTAAACAGGCTGGTCTGTCCTTTGGTTTTGGGGATT
